# Rheumatic Heart Disease in Pregnancy: New Strategies for an Old Disease?

**DOI:** 10.5334/gh.1079

**Published:** 2021-12-20

**Authors:** Geraldine Vaughan, Angela Dawson, Michael Peek, Karen Sliwa, Jonathan Carapetis, Vicki Wade, Elizabeth Sullivan

**Affiliations:** 1 Central Queensland University, AU

**Keywords:** Rheumatic heart disease, Pregnancy, First Nation peoples, Inequity

## Abstract

RHD in pregnancy (RHD-P) is associated with an increased burden of maternal and perinatal morbidity and mortality. A sequellae of rheumatic fever resulting in heart valve damage if untreated, RHD is twice as common in women. In providing an historical overview, this commentary provides context for prevention and treatment in the 21 st century.

Four underlying themes inform much of the literature on RHD-P: its association with inequities; often-complex care requirements; demands for integrated care models, and a life-course approach. While there have been some gains particularly in awareness, strengthened policies and funding strategies are required to sustain improvements in the RHD landscape and consequently improve outcomes.

As the principal heart disease seen in pregnant women in endemic regions, it is unlikely that the Sustainable Development Goal 3 target of reduced global maternal mortality ratio can be met by 2030 if RHD is not better addressed for women and girls.

## A History of Broken Hearts: Rheumatic Heart Disease in Pregnancy

In 2021, a young pregnant Aboriginal woman with rheumatic heart disease (RHD) was told *‘…didn’t know we had that (RHD) anymore.’* Yet this preventable disease continues to devastate Aboriginal and Torres Strait Islander communities. Despite being a high-income country overall, First Nation Australians experience some of the highest documented rates of RHD in pregnancy in the world, particularly remote communities in Northern Australia which face significant disadvantage and lack of services. However, overall, low-resource regions sustain the global burden of RHD: 80% of people with RHD live in low-and middle-income countries (LMICs) [1].

A sequellae of acute rheumatic fever (ARF) that results in lasting heart valve damage if untreated [2], RHD is twice as common in women, most likely associated with several factors including increased risk of autoimmune disease, accelerated progression of mild RHD during pregnancy and increased exposure to StrepA infection. What is the epidemiology of this ancient disease and how has it been addressed in pregnancy through the millennia? In providing an historical overview, this commentary provides context for the prevention and management in the 21^st^ century. It considers how lessons learnt in the 19^th^–20^th^ century are of relevance today.

While health systems across the centuries and across the world may differ enormously according to developments in knowledge, technologies, economies, and social structures, four underlying themes inform much of the literature of RHD in pregnancy (RHD-P): the association of RHD with inequities and poverty; recognition of often-complex care requirements during pregnancy; demands for integrated care models to optimise maternal and perinatal outcomes, and a life-course approach to RHD for women and girls.

## Risk Through the Millennia for Pregnant Women with RHD

Nearly 2,500 years ago, Hippocrates discussed the classic symptoms of rheumatic mitral stenosis:

*‘Harpalida’s sister, in the fourth or fifth month of her pregnancy, had watery swellings in her legs, swellings in the hollows of her eyes, and her whole body puffed up… Sometimes she was so near to suffocation that she was obliged to sit up in her bed without being able to lie down; and if she tried to sleep it was in a sitting position. Yet there was not much fever. For a long time the foetus did not move, as if it were dead.…’ Epidemics VII VI (Littre, 1839–1861; Clifton, 1734) in* [3].

In the 1800s, several writings noted poor outcomes of pregnancy complicated by cardiac disease, which was overwhelmingly caused by RHD in the overcrowded urbanised centres of the United Kingdom (UK), Europe and North America. The death of a young woman in labour due to (rheumatic) mitral stenosis was a key driver in Scottish cardiologist James Mackenzie’s work to improve the clinical management of heart disease [4].

*I discovered that [a young woman who had a miscarriage] had a presystolic murmur… I had a vague notion that mitral stenosis was a serious embarrassment to the heart in pregnancy, but of the source of danger and its nature I had no knowledge. The woman …[later] returned … seven months pregnant, and with a considerable oedema of the legs. … As the labour proceeded little progress was made; the suffering was very great, and there was marked distress in breathing… after many hours of suffering she died undelivered* [5].

Around the same time, obstetrician Angus Macdonald wrote his defining textbook on cardiac disease in pregnancy. There was little on congenital heart disease: in the nineteenth century, women rarely survived to adulthood. Much of his focus was on mitral stenosis of rheumatic origin:

*We have thus nine cases out of the fourteen [pregnancies], or 64.4%, fatal, which indicates a tendency to death in the combination of mitral stenosis with pregnancy which is surely sufficiently grave…* [6].

However, despite such outcomes, clinicians such as McKenzie, Macdonald and others did not proscribe pregnancy altogether [7], but counselled according to lesion and clinical status, developing a risk stratification in clinical care, concluding that…

*These good results encourage us to believe that judicious and skilful management of similar cases, both throughout the pregnancy and at delivery, might greatly lessen the risks associated with lesions* [of mitral stenosis] [6].

Macdonald also mused on the ethical responsibilities of obstetricians to advise women on pregnancy choices (with marriage seen as a proxy), which he argued were largely unconsidered:

*I know of no English writer who has striven to put obstetricians in a position to answer with intelligence the question that is every now and again asked of us by a patient who knows she has heart disease, — Should I marry? or do I run great risk in marrying? … [a] great amount of ignorance exists* [6].

In the late 1940s, the first known longitudinal study of maternal outcomes used the recently developed New York Heart Association (NYHA) functional classification of cardiac disease based on clinical severity and prognosis to mark the progression of cardiac disease in 169 pregnant women [8,9,10,11,12,13].

This article drew on others’ research to conclude that RHD of itself was not an indication for caesarean section, counselling that this should be avoided unless indicated by obstetric reasons or severe cardiac compromise [10,12,13,14].

## ‘*The Cramping Effects of Too-Rigid Specialism*’: Care Across Disciplines

Integrated care across specialisations was a critical aspect of developing strategies to address the risk for women with RHD-P. There had been calls for collaborative cardiac care for pregnant women from the late nineteenth century. Macdonald admonished his British obstetric colleagues to devote more attention to cardiac disease in pregnancy … *‘commensurate with its importance’* in order to better *‘…differentiate the effects of the special cardiac lesions, and to define in any way their individual bearings upon either pregnancy or parturition.’* Obstetricians’ *‘rigid specialism’* he declared, compromised effective management of women under their care, and led to much of the *‘disfavour and affected hauteur with which obstetrical medicine has frequently been treated by pure physicians and surgeons’* [6].

Good nursing care was considered ‘*…likely to be better rewarded … in warding off the exciting causes of pulmonary disturbances in connection with pregnancy complicated with various cardiac lesions’* [6], but disciplinary references outside cardiac and obstetric specialisations were largely absent.

However, the next half-century did not bring much evidence of better awareness nor improved collaborative care. Some forty years later, McKenzie observed the knowledge of cardiac disease among obstetric physicians [5]. He spoke of the fear of consequences for women and the need to provide informed and educated advice:

*These disastrous [cardiac] happenings surround a natural process with dread and mystery, for the dread is aggravated by the fact that the source of danger is not clearly realised. As a result all sorts of signs are looked upon with suspicion—signs innocent as well as signs grave. Needless to say, this obscurity does a great deal of harm. Many women are subjected to unnecessary alarms and restrictions when pregnant ; others have to suppress the natural desire of motherhood…* [5].

The collaborative models that Macdonald and McKenzie called for were being slowly developed. A Scottish joint cardiac-obstetric clinic established in 1928 saw cardiac deaths as a percentage of cardiac cases (94% with underlying rheumatic pathology) drop from 6.3 to 0.9% by 1947 [10].

Hamilton described similar trends in North American centres. When a cardiac clinic commenced at Boston Lying-In Hospital in 1921, the maternal mortality rate was 20% (93% due to RHD), which dropped to under 5% in the ensuing three years [15]. Review and treatment (rather than the RHD condition per se) was among the largest prognostic factor in this study, with regular antenatal review during pregnancy stressed as an essential component of care [15].

A 1936 Canadian study called for the… *‘establishment of a combined prenatal and cardiac clinic which has permitted personal consultations between cardiologist and obstetrician and a continuity of observation of all heart cases’* [16].

From the 1930s, similar studies in London and Dublin found that, with adequate collaborative cardiac-obstetric care the need for medically advised terminations of pregnancy was reduced significantly and outcomes improved [17,18]. McIlroy (the first UK female obstetrician and medical professor), in recognising the overwhelming burden of RHD in cardiac pathology, detailed the mutual benefits of obstetric-cardiac collaboration, including earlier diagnosis of cases [17]. A review of women with RHD-P at two UK sites over 28 years (1942–1969) concluded that *‘routine medical examinations at about 10, 15, and 20 years of age with appropriate management would render pregnancy virtually safe for every patient with rheumatic heart disease’* [19].

However, such outcomes were challenged by a lack of interdisciplinary collaborative approach to care and gaps in health system support which persists to the current day:

*Another difficulty arises from the different way in which the cardiologist and the obstetrician regard the problem. The cardiologist considers the pregnancy as complicating the pre-existing heart disease, but the obstetrician considers that his [sic] patient’s pregnancy is complicated by her cardiac lesion. Another cause for ignorance is that the obstetrician rarely studies his patients once the puerperium has been successfully passed, and if further pregnancies do not ensue he may never see the patient again. On the other hand, the cardiologist rarely follows the same patient through several pregnancies unless he is particularly interested in the subject, and his opportunities for observing closely the heart in normal pregnancy are few* [13].

## The Shifting Global Burden of RHD

The epidemiology of RHD generally mirrors the evolution of nation economies and associated changing health profiles. By the 1960s, the overall prevalence of RHD was waning in high-income countries (HIC) [20,21,22,23,24]. Studies of maternal morbidity and mortality trends in HIC began noting the increasing proportion of women with congenital heart disease compared to a decrease in RHD [25,26,27] during pregnancy.

Unsurprisingly, this decline was not shared by low-income countries: in the 21^st^ century, the global burden of disease has shifted to (in particular) regions such as Oceania, South Asia, central sub-Saharan Africa [28,29,30], the Caribbean and Latin America [31], with a correspondingly high burden in pregnancy [32,33,34,35,36,37,38,39].

Reflecting the association of RHD with inequities, the reduced burden of disease has varied *within* HICs [40,41,42,43,44,45], with an increasing (and persistent) trend among First Nations [2,40,46,47,48], as well as migrants from low-resource countries [2,49,50].

The early 2000s saw a shift in policy and political will related to RHD, no doubt galvanised by increasing advocacy and locally-driven initiatives, and supported by increasing evidence of the global burden of RHD [1,50,51,52,53,54,55,56,57,58]. Several LMICs have formed national programs dedicated to RHD prevention and control [59,60,61,62], with a 2013 World Heart Federation (WHF) goal of a 25% reduction in premature deaths from RF/RHD among individuals aged <25 years by the year 2025 [63]. An important development has been the increased focus on (and working with) people living with RHD and frontline health workers delivering essential RHD services [64,65,66,67,68,69,70].

The need to situate RHD strategies within the context of maternal health and access to services was of critical note in the 2018 World Health Organization’s first global policy on ARF/RHD [71]. Improved access to reproductive health services for women with RHD and other non-communicable diseases (NCD) is one of the seven key priority actions called for in a roadmap to eliminate RF and eradicate RHD in Africa [52,72].

## *Why So Complicated?* Barriers to Optimal Care for Women with RHD

While challenges to optimal care in the RHD and pregnancy landscape can be broadly categorised –access to multidisciplinary health services, continuity of care, geographical distances, health workforce resourcing and education (particularly in primary care), sustained support for community-driven initiatives [56,68,69,70,73], fragmented health information silos [74] – they form a complex matrix of contributing factors that can impact directly and on each other. Underpinning each of these are the policies, governance, political will and practical decisions that shape the promotion – or hindering – of culturally safe and effective models of care.

The additional risks in pregnancy of a compromised heart through (possibly undiagnosed) RHD further escalates where anticoagulation and interventional treatments are required, and highlights the necessity for a life-course approach to care including preconception (addressing needs for young people transitioning to adulthood), pregnancy (including early and regular antenatal reviews), the postpartum and interpregnancy periods.

Yet, the compelling need to redress the neglect of RHD and RHD-P in public health and funding arenas persists. Even ignoring the critical social justice issues of RF/RHD, its associated fiscal burden demands significantly better support [75,76,77]: it costs USD5 to treat a sore throat, compared to the lifetime burden associated with valvular surgery upwards of USD29,000 [56,62,78,79]. The maternal and perinatal costs of the burden of RHD are exponentially higher [80], particularly when the indirect costs of economic impact are taken into account – family, community, work – are taken into account.

## *Work with Us*: Integration and Collaboration

While specific approaches to best-practice care for women with RHD in pregnancy will vary according to country, culture and resources; general principles emphasise the continuity of care, underpinned by collaborative partnerships across sectors [34,45,48,64,70,81,82].

Of course, many principles of care – preconception woman-centred care, a multidisciplinary approach to service development and care guidelines – are equally applicable to women with all-cardiac disease [82,83]. However, as a sentinel disease of inequity, RHD exemplifies the ‘causes of the causes’ that underpin compromised health [84], are particularly relevant in pregnancy.

Improved maternal and perinatal outcomes afforded by multidisciplinary models, particularly joint obstetric–cardiac care for women with complex disease are evident across the spectrum of low-resource [81,85], and high-income settings [45,82,86].

## Conclusion

This historical perspective illustrates some of the themes and issues central to care pathways for women with RHD-P: diagnosis, pregnancy planning, risk assessment, often-complex care needs, continuity of care and access to woman-centred integrated health services. These physical and structural imperatives are against a backdrop of often-resource challenged environments that test optimal care and outcomes for mother and baby.

The 19^th^ century strategies that called for collaborative models and cross-sectoral partnerships are as relevant today. While there have been some gains particularly in awareness of the impact of RHD in pregnancy, we need the societal and political will, sound policies and funding strategies required to support sustained and meaningful improvements in the RHD landscape and consequently improved care in pregnancy.

As the principal heart disease seen in pregnant women in endemic regions, it is unlikely that the Sustainable Development Goal 3 target of reducing the global maternal mortality ratio can be met by 2030 if RHD is not better addressed for women and girls.
